# Dangers of the Cold Bath

**Published:** 1883-04

**Authors:** 


### DANGERS OF THE COLD BATH.

Alluding to a recent case of death of an old gentleman, caused by a morning cold bath, the *Medical Press and Circular* says: "The great mistake that is usually committed in regard to it is the error of never raising the temperature of the water from that of the surrounding air. In very cold weather the bath, even when exposed over night in the bedroom, will often be lower than 45 degrees, and where water is brought straight from the main or well it may be even 10 or 15 degrees lower. Only the strongest constitutions can derive benefit from the shock produced by application of a liquid 60 to 70 degrees colder than the body to its surface, and it is very questionable if it is ever attended with permanently good results. Reaction may be afterward complete; but there is always the risk of sudden danger from the condition of the body being temporarily such as to prevent immediate reaction. In such cases very serious accidents are possible, and this last instance of death may perhaps be regarded as an example in point. A temperature of from 40 to 50 degrees is quite cold enough for any person to submit himself to. This allows for a difference of between 40 and 50 degrees in the heat of the body and that of the bath—amply sufficient to produce all the benefits desirable from it—and it would be well for all if these extremes were never exceeded."

---

The London *Lancet* says that muscarine, the active poison of mushrooms, is directly antagonized by atropia. A trace of muscarine placed upon a frog's heart completely arrests its motion; a drop of atropia will start it up again, although it may have remained motionless for four hours. In human beings poisoned by mushrooms one minim of atropia, administered hypodermically at intervals, effects a complete cure.

Pure and good milk is a necessity in almost every family. It may come into the house in a wholesome condition, yet there is ever the danger that it will become tainted with the sewer gas from closets, or even with the dust carried by drafts through sleeping or living rooms. There can be no doubt that while milk is one of the best and most palatable of foods in summer, it must be carefully guarded from the farm to the table, or it will prove a potent vehicle of disease.

